# Leveraging Resource Centers for Strengthening Immunization Supply Chain

**DOI:** 10.7759/cureus.58966

**Published:** 2024-04-25

**Authors:** Snehil K Singh, Gajendra K Gupta, Deepika Agrawal, Syed Hasan N Zaidi, Jyoti Batra, Lokesh Sharma, Sumeet Juneja, Krupal J Joshi, Ghanshyam Sethy, Dereje A Haile, Sabin Syed

**Affiliations:** 1 Community Medicine, Santosh, Deemed To Be University, Ghaziabad, IND; 2 Biochemistry, Santosh, Deemed To Be University, Ghaziabad, IND; 3 Immunization Supply Chain, United Nations Children's Fund (UNICEF), New Delhi, IND; 4 Immunization Supply Chain, International Consultant, Public Health, Delhi, IND; 5 Community and Family Medicine, AIIMS Rajkot-Institute of National Importance, Rajkot, IND; 6 Child Health, United Nations Children's Fund (UNICEF), Lilongwe, MWI; 7 Health System Strengthening, United Nations Children's Fund (UNICEF) Regional Office for East and Southern Africa, Nairobi, KEN; 8 Health System Strengthening, Primary Healthcare Capacity Building, Lilongwe, MWI

**Keywords:** vaccine equity, immunization agenda 2030, cold chain management, health system strengthening, vaccine logistics management, capacity building, vaccine management, center of excellence, resource center, immunization supply chain

## Abstract

The efficacy of immunization programs is critically dependent on robust supply chain management, a complex challenge exacerbated by expanding program scopes and evolving vaccine technologies. This comprehensive review underscores the pivotal role of Resource Centers in fortifying the immunization supply chain, presenting a paradigm shift toward enhanced national and global health outcomes. Through a detailed examination of their key activities, the article elucidates how these centers catalyze improvements across various facets of supply chain management - from the integration of suitable technology technologies and specialized training programs to the development of sustainable models and advocacy for policy prioritization. This further explores the multifaceted challenges these centers confront, including funding constraints, capacity building, and infrastructural gaps, alongside the burgeoning opportunities presented by new vaccine introductions, donor interest in health system strengthening, and the potential for broadened scope beyond immunization. By weaving together examples of existing centers worldwide, the review highlights their contributions towards optimizing vaccine logistics, enhancing data management, and ultimately achieving Sustainable Development Goal 3. The insights provided offer valuable guidance for planning and sustaining resource centers, positioning them as indispensable allies in the global pursuit of universal immunization coverage.

## Introduction and background

Immunization, the bedrock of global public health, has been instrumental in averting innumerable deaths and curbing the spread of infectious diseases [1]. Central to the success of immunization programs is the often-overlooked realm of the immunization supply chain [2]. This intricate network spans the entire journey of vaccines - from production facilities to the arms of recipients - and plays a pivotal role in safeguarding vaccine potency and efficacy.

Despite the tremendous strides made in global immunization efforts, supply chain management remains an enduring challenge [3]. The crux of this challenge lies in the need to maintain vaccines under recommended temperature and logistical conditions, a task that becomes increasingly complex as immunization programs expand and diversify.

The WHO's Effective Vaccine Management (EVM) assessments from 2009 to 2020 have highlighted critical areas in the global immunization supply chain that require robust intervention. These include vaccine distribution, with median scores consistently below the EVM standard of 80% at all supply chain levels, and temperature monitoring, which is often inadequate, particularly at the lowest distribution and service point levels. Additionally, there are significant deficits in information systems, essential for EVM. These challenges manifest in operational inefficiencies and increased vaccine wastage, as evidenced by less than 20% of countries conducting a systematic temperature monitoring study in the past five years, and many facilities unable to fully satisfy all vaccine requests due to stock mismatches or insufficient stock levels. This resource center can strategically address these gaps by implementing advanced training programs, supporting the adoption of modern information systems, and facilitating the integration of technology to enhance real-time data availability and decision-making in vaccine logistics [4].

Recognizing the imperative of robust supply chain management, a groundbreaking approach has emerged: the establishment of Resource Centers or Centers of Excellence dedicated to the optimization of immunization supply chains [5]. These centers embody not just expertise but also a commitment to advancing the science of immunization supply chain for the betterment of global public health.

Concept of resource centers

Resource centers specializing in immunization supply chain represent a paradigm shift in global health strategy. Whether operating at a national or international level, these centers serve as focal points for expertise, knowledge dissemination, training, and support, transcending geographical boundaries to bolster immunization programs worldwide [6].

## Review

Landscape of similar centers worldwide

Across the globe, numerous centers of excellence and resource centers have emerged, each committed to addressing critical healthcare challenges, including but not limited to the optimization of the immunization supply chain and other health resources [7]. These centers serve as invaluable hubs for knowledge dissemination, capacity building, and collaboration. Table 1 shows a brief landscape of similar centers worldwide.

**Table 1:** Landscape of similar centers worldwide

National Cold Chain and Vaccine Management Resource Centre (NCCVMRC), India, Location: Delhi, India	*Swiss Vaccine Supply Chain Center of Excellence (VSC CoE), Switzerland, Location: Switzerland*	Regional Centre of Excellence for Vaccines, Immunization, and Health Supply Chain Management, Rwanda (RCE-VIHSCM), Location: Rwanda	International Center for Diarrhoeal Diseases Research, Bangladesh (icddr,b), Location: Bangladesh	LOGIVAC Bénin, Location: Benin
*Overview:* NCCVMRC is a renowned institution specializing in optimizing the immunization supply chain. It offers a comprehensive range of services, including training, research, consultancy, and technology development [7]. NCCVMRC has made significant contributions to enhancing vaccine logistics and distribution in the South Asian region [8].	*Overview*: Switzerland hosts the VSC CoE, a globally recognized institution focusing on vaccine supply chain management. It collaborates with international organizations, governments, and industry stakeholders to advance vaccine distribution networks, improve logistics, and ensure vaccine quality and safety. The VSC CoE's expertise extends to supporting immunization programs worldwide [9].	*Overview*: RCE-VIHSCM aims to professionalize and modernize immunization supply chain across East Africa. Its mission is to provide basic and advanced training for health supply chain management and serve as a regional hub for introducing innovative procedures [10]. The center offers various training programs, conducts research, and operates a resource center with a knowledge database. It collaborates with partners such as the University of Rwanda and receives funding from entities like the German government, GAVI, and UNICEF.	*Overview*: icddr,b envisions a world where more people survive and enjoy healthy lives. Its mission is to solve public health problems through innovative scientific research. It conducts research to inform national policies, develops interventions, operates field sites, publishes research findings, offers training programs, provides clinical services, and offers laboratory sciences and services [11]. It maintains numerous international collaborations and partnerships with organizations like USAID and UNICEF.	*Overview*: LOGIVAC Bénin shares the mission of professionalizing and modernizing immunization supply chain in East African countries, with a focus on training for health supply chain management [12]. The center provides training, equips health zones with solar-powered refrigerators, and collaborates with the Regional Institute of Public Health in Ouidah. Funding is provided by the Bill & Melinda Gates Foundation

Such Centers of Excellence serve as pillars for research, training, and collaboration in healthcare supply chain management. Their contributions to improving vaccine logistics, quality, and distribution have a significant impact on public health. Understanding their organizational structures, funding mechanisms, and core activities offers valuable insights for planning and establishing similar institutions dedicated to advancing immunization supply chain management [13].

Need for resource centers

The growing complexity of vaccine supply chains, coupled with the expanding array of vaccines, necessitates a concerted effort to ensure their effective management. Immunization programs are confronted with challenges such as temperature-sensitive vaccines, diverse vaccine portfolios, and the need for real-time data management [14]. These challenges transcend national boundaries and require collaborative solutions.

Furthermore, the COVID-19 pandemic has underscored the criticality of a robust supply chain for vaccines and medical commodities [15]. As the world grapples with the logistical intricacies of vaccine distribution on an unprecedented scale, the need for expertise in supply chain management has never been more apparent.

In this context, the establishment of Resource Centers dedicated to the immunization supply chain becomes not just a strategic choice but an imperative. These centers offer a nexus for collaboration, knowledge-sharing, and innovation, with the potential to transform the landscape of global immunization supply chain management [16].

Tangible benefits of establishing resource centers for immunization supply chains

The establishment of Resource Centers for the Immunization Supply Chain catalyzes significant advancements in public health infrastructure, directly contributing to the efficacy, reach, and resilience of global immunization efforts [17].

The establishment of International Resource Centers for Immunization Supply Chains aligns closely with the Immunization Agenda 2030's Strategic Priority 6, which emphasizes “Supply and Sustainability,” as well as the Global Alliance for Vaccines and Immunization (GAVIs) second goal to “Strengthen Health Systems to Increase Equity in Immunization.” These centers significantly enhance the efficiency and resilience of immunization supply chains by integrating cutting-edge technologies and best practices. This strategic alignment ensures the effective distribution of vaccines, especially in underserved regions, thereby supporting global efforts to increase equitable access to immunization and fortify health systems as outlined by both IA2030 [18] and GAVI [19].

The tangible outcomes of these centers' activities manifest in various dimensions, as highlighted below.

Improved Immunization Supply Chain Efficiency

The streamlined processes and innovations introduced by these centers lead to markedly more efficient immunization supply chains. Enhanced logistic strategies and the implementation of best practices facilitate the swift and effective distribution of vaccines, ensuring timely immunization coverage across diverse geographies [20].

Reduction in Vaccine Wastage

A critical outcome of the optimization efforts by these centers is the significant reduction in vaccine wastage. Through improved cold chain management and inventory practices, vaccines are preserved in optimal conditions, minimizing losses due to expiration or improper storage [21].

Optimization of Cold Chain Equipment

The deployment and better utilization of advanced cold chain equipment, guided by the research and recommendations from these centers, ensure vaccines maintain their potency from manufacture to administration. This optimization contributes to the overall effectiveness of immunization programs and ensures the right equipment at the right place [22].

Inclusion of Newer Technology

Resource Centers spearhead the incorporation of cutting-edge technology into immunization supply chains, from temperature monitoring devices to data analytics platforms. These technological advancements enable real-time tracking and management of vaccine stocks, enhancing the responsiveness of immunization programs to emergent needs [23].

Skill Enhancement of the Workforce

By providing targeted training and capacity-building programs, these centers ensure that the workforce managing the immunization supply chain is skilled, knowledgeable, and capable of employing the latest practices and technologies. This skill enhancement directly impacts the quality and effectiveness of vaccine management practices [6].

Enhanced Immunization Supply Chain and Vaccine Logistics Data Management

One of the critical benefits stemming from the activities of Resource Centers is the significant enhancement in data management capabilities within immunization supply chains. These centers promote the use of sophisticated data analytics tools and systems, enabling real-time tracking, forecasting, and management of vaccine inventories [24]. Improved data management leads to optimized vaccine logistics, ensuring that vaccines are delivered where and when they are needed most, thus maximizing reach and impact.

Sustainability of immunization resource centers: a path to self-reliance

Sustainability is the linchpin of any successful global health initiative, ensuring that its impact endures beyond initial funding cycles. Resource Centers for the immunization supply chain are no exception. These centers can adopt various strategies and mechanisms to become self-sustainable while continuing to provide essential support to immunization programs [25]. Here, we outline some key approaches, coupled with real-world examples, to illustrate the path to sustainability.

Revenue Diversification

Training programs: Centers can generate revenue by offering training programs to immunization professionals [26]. For example, the Sabin Vaccine Institute's Boost program conducts training sessions on supply chain management and sustainability, charging a fee for participation.

Consultancy services: Centers can provide consultancy services to governments, organizations, and vaccine manufacturers. The Pan American Health Organization (PAHO) offers consultancy services on vaccine procurement, logistics, and supply chain management to countries in the Americas [27].

Public-Private Partnerships

Centers can collaborate with private-sector partners for financial support. UNICEF's partnership with GAVI, the Vaccine Alliance, includes contributions from corporate partners such as UPS and Novo Nordisk to strengthen immunization supply chains [28].

Government Ownership and Funding

Centers can work closely with governments to gradually transition financial responsibility for the center's operations. For instance, the South African National Department of Health supports the National Health Laboratory Service (NHLS), which provides laboratory services for vaccine-preventable diseases [29]. Governments can allocate a portion of their health budgets to sustain resource centers. The Ethiopian government, through its Health Transformation Plan, funds the Ethiopian Pharmaceuticals Supply Agency (EPSA) to manage vaccine supply chains [30].

Income-Generating Activities

Centers can engage in research and development activities, leading to innovations that can be monetized. PATH's Vaccine Development Program conducts research on new vaccines and technologies while collaborating with industry partners for funding [31].

Endowment Funds

Centers can create endowment funds, where a portion of investment return supports ongoing operations. The Gates Foundation established an endowment for the GAVI, ensuring long-term sustainability [32].

Partnerships With Academia

Centers can partner with academic institutions to offer joint programs, such as master's degrees or certifications in supply chain management [33]. The Liverpool School of Tropical Medicine collaborates with the University of Ghana to offer a Master of Public Health program with a focus on immunization.

Leveraging International Initiatives

Centers can seek support from international funding initiatives. The Global Fund to Fight AIDS, Tuberculosis, and Malaria provides funding for strengthening health systems, including supply chain management [33]. Similarly, GAVI through UNICEF has supported National Cold Chain and Vaccine Management Resource Centre for the effective delivery of vaccine cold chain and supply chain services in India.

Cost-Recovery Mechanisms

Countries can introduce small levies on vaccines to support supply chain strengthening. The GAVI COVAX Advance Market Commitment (AMC) uses a similar approach to fund COVID-19 vaccine distribution in low-income countries [34].

Donor Collaboration

Centers can collaborate with donors to pool resources and streamline support. The World Bank's International Development Association (IDA) combines resources with other donors to support health programs in low-income countries [35].

Monitoring and Evaluation

Regularly assess the impact and cost-effectiveness of center activities to demonstrate value to stakeholders and potential funders. The WHO's Expanded Programme on Immunization (EPI) conducts impact evaluations to guide program improvements [36].

Sustainability is not a one-size-fits-all endeavor, and the specific approach taken by each resource center will depend on its context, resources, and goals. By diversifying revenue sources, fostering partnerships, and engaging governments, resource centers can transition from dependency on external funding to self-reliance, ensuring their long-term contribution to strengthening global immunization supply chains.

Key activities of resource centers for immunization supply chains

Resource Centers for Immunization Supply Chain perform critical functions aimed at enhancing the effectiveness, reach, and sustainability of immunization programs globally. These centers engage in a diverse range of activities:

Specialized Training and Professional Development

Resource Centers design and deliver specialized training programs for health workers and supply chain managers, focusing on the latest methodologies in vaccine logistics and cold chain management. These programs are crucial for building a knowledgeable workforce capable of addressing the complexities of global immunization supply chains [25].

Technological Innovation and Integration

Driving innovation is a cornerstone activity of these centers, with a particular emphasis on developing and deploying new technologies to streamline supply chain operations. This includes advanced cold chain equipment, temperature monitoring solutions, and logistics management systems [23], which are vital for maintaining vaccine integrity and optimizing distribution.

Technical Assistance and Consultancy

Providing targeted technical assistance to countries and health organizations, Resource Centers help design and implement efficient supply chain solutions. This hands-on support covers a wide range of needs, from supply chain assessments to the execution of strategic improvements, ensuring that immunization programs can overcome local and regional challenges [20].

Research, Data Analysis, and Policy Development

Through rigorous research and comprehensive data analysis, these centers identify best practices and areas for innovation within immunization supply chains. Their findings inform both global and local policy development, helping to shape strategies that strengthen supply chain resilience and effectiveness [22].

Emergency Preparedness and Response Planning

Center plays a critical role in preparing for and responding to public health emergencies that impact vaccine supply and distribution. By developing contingency plans and coordinating with global health bodies, they ensure that immunization efforts can quickly adapt to crises, maintaining the availability of potent vaccines and preventing outbreaks [37].

Facilitation of Global and Regional Collaboration

Acting as hubs for collaboration, Resource Centers bring together various stakeholders from government, non-governmental organizations, academia, and the private sector. This collaborative environment fosters the exchange of knowledge, resources, and best practices, amplifying the impact of collective efforts toward immunization goals [38].

Advocacy for Immunization Supply Chain Prioritization

Through advocacy efforts, these centers highlight the critical importance of a robust immunization supply chain to global health leaders and policymakers. Their work ensures that supply chain strengthening remains a priority in global health agendas, securing the necessary support and resources for continuous improvement [17].

Challenges

Funding Constraints

Establishing and sustaining resource centers requires substantial financial investments. Securing consistent funding sources can be challenging, and over-reliance on donor funding may lead to uncertainties [39].

Capacity Building

Developing a skilled workforce, especially in resource-limited settings, can be a significant challenge [40]. Providing ongoing training and retaining talented professionals is crucial.

Infrastructure and Technology Gaps

Many countries, particularly in low-resource settings, face infrastructure and technology limitations. Ensuring the availability of reliable cold chain equipment and technology can be a hurdle.

Political and Regulatory Challenges

Immunization programs are subject to political changes, and regulations can vary significantly between countries. Resource centers may need to navigate complex political landscapes [41].

Health Systems Strengthening

Immunization supply chains are embedded within broader health systems. Addressing supply chain challenges often requires strengthening the overall health system, which can be a complex and time-consuming process [42].

Adaptation to Rapid Changes in Vaccine Demand and Supply Dynamics

The global health landscape, particularly in the context of pandemics or outbreaks, can shift rapidly, affecting vaccine demand and supply logistics. Centers must remain adaptable and proactive in forecasting, planning, and responding to these changes to ensure vaccine delivery remains uninterrupted and effective [43]. This challenge encapsulates the need for agility in operations and strategy, a critical aspect of managing the supply chain in an unpredictable global health environment.

Opportunities for resource centers for immunization supply chains

The landscape of global health, particularly immunization supply chains, presents numerous evolving opportunities. These opportunities not only address current challenges but also pave the way for transformative improvements in vaccine delivery systems worldwide:

Adapting to Innovations in Vaccine Delivery

The advent of new vaccine technologies, such as microneedle patches and heat-stable vaccines, presents an opportunity for Resource Centers to lead in the adoption and integration of these innovations [44]. These technologies can simplify vaccine administration and storage, significantly enhancing the reach and efficiency of immunization programs.

Expansion and Modernization of Cold Chain Infrastructure

The introduction of new vaccines necessitates the expansion and modernization of cold chain infrastructure. Solutions like Solar Direct Drive (SDD) refrigerators and solar electrification of health facilities represent key areas where centers can drive improvements, ensuring vaccines are stored and transported under optimal conditions [45].

Leveraging Interest from Donor Agencies in Health System Strengthening

The growing interest of donor agencies in funding health system strengthening, including immunization supply chains, offers resource centers an opportunity to secure resources for innovation and capacity building [46]. This support is crucial for the sustainable improvement of vaccine delivery services.

Enhancing Global Coordination and Integrated Health Systems

Increasing global coordination and the move towards an Integrated Health System Approach present opportunities for resource centers to foster collaboration across borders and health sectors. This integrated approach is essential for creating resilient health systems capable of responding to emerging health challenges effectively [47].

Promoting Evidence-Based Decision Making

The engagement of governments, donors, UN agencies, and program managers in evidence-based decision-making underscores the importance of data and research-driven strategies. Resource centers can play a pivotal role in generating and disseminating evidence to inform policy and operational decisions [48].

Exploring Self-Sustainable Models

The demand for self-sustainable models offers an opportunity for resource centers to innovate in financial and operational strategies. Developing mechanisms for cost recovery and efficiency can ensure the long-term viability and impact of these centers [49].

Expansion beyond immunization

The success and impact of initiatives like the Immunization Supply Chain Resource Center serve as a compelling precedent for the future expansion of similar initiatives in the broader domain of healthcare resource management [50]. With the growing importance of robust healthcare systems globally, extending the concept of resource centers to other critical areas such as blood bank management, oxygen supply chain management, Health Care Waste Management and the management of various medical equipment is not only feasible but also highly desirable.

The expansion of initiatives like resource centers beyond immunization supply chain management holds immense potential to strengthen healthcare systems worldwide. By addressing critical needs in such areas, these initiatives can further advance global health outcomes, promote sustainability, and serve as valuable models for effective resource utilization in the healthcare sector.

## Conclusions

In conclusion, the establishment of Resource Centers for Immunization Supply Chain represents a pivotal step in the global commitment to achieving universal immunization coverage with potent vaccines. These centers serve as beacons of knowledge, expertise, and collaboration, bringing together nations, partners, and professionals to address the complex challenges of vaccine supply chain management. The need for such resource centers is underscored by the increasing demand for vaccines, the expansion of immunization programs, and the imperative to reach every child, regardless of their geographic location. Similar centers across the world have demonstrated their value by providing technical assistance, capacity building, and innovative solutions to enhance the efficiency and effectiveness of immunization supply chains. The benefits of these resource centers are multifaceted. They empower countries to optimize their supply chains, improve vaccine availability, reduce stockouts, and ultimately save lives. By fostering global collaboration, sharing best practices, and advocating for immunization, these centers contribute to the achievement of Sustainable Development Goal 3 - ensuring healthy lives and promoting well-being for all.

Sustainable funding, private sector engagement, and policy advocacy are essential for the longevity and success of these centers, which have shown potential to become self-sustaining. Their work in capacity building, research, policy formulation, emergency response, and informed decision-making is vital for strengthening health systems in totality. Despite challenges like funding limitations and regulatory complexities, these centers are catalysts for innovation and sustainable solutions. They symbolize a global pledge to equitable immunization services, ensuring no child is left behind. As the world moves towards full immunization coverage, these resource centers are key allies in fostering a healthier, more equitable future for all children.
